# Somatic *NLRP3* mosaicism in patients with “mutation-negative” CAPS: insights from a single centre UK cohort

**DOI:** 10.3389/fped.2025.1598748

**Published:** 2025-06-05

**Authors:** Sonia Melo Gomes, Juan I. Arostegui, Ana Mensa-Vilaro, Ebun Omoyinmi, Ying Hong, Dara McCreary, Dorota Rowczenio, Philip Hawkins, Paul Brogan

**Affiliations:** ^1^ Department of Rheumatology, Great Ormond Street Hospital, London, United Kingdom; ^2^ Department of Immunology, Hospital Clinic, Barcelona, Spain; ^3^ Department of Infection, Immunity and Inflammation, UCL GOS Institute of Child Health, London, United Kingdom; ^4^ National Amyloidosis Centre, Royal Free Hospital, London, United Kingdom

**Keywords:** mosaicism, CAPS, NLRP3, NGS—next generation sequencing, mosaicism distribution, mosaicism stability

## Abstract

**Introduction:**

Knowledge about mosaicism in cryopyrin-associated periodic syndromes (CAPS) has expanded significantly with the use of next generation sequencing technologies. The aim of this study was to assess the contribution of mosaicism in a paediatric cohort of patients with a clinical diagnosis of CAPS and no *NLRP3* mutations identified through conventional DNA sequencing.

**Methods:**

Mosaicism was assessed by amplicon-based deep sequencing (ADS) on DNA extracted from different tissues overtime. Targeted gene panels (TGPs) and whole-exome sequencing (WES) were used for comparison of detection methods.

**Results:**

In 40% (4/10) of the cohort a post-zygotic *NLRP3* mutation leading to somatic mosaicism was found by ADS. Three of the detected *NLRP3* mutations had been previously described only in somatic form and one both as germline and somatic. Mean mutant allelic frequencies (MAF) at diagnosis varied between 3.1-14.5% in whole blood, with all mutations being present in other tissues tested. In 3 patients, mosaicism was evaluated over time in whole blood, with results confirming mosaicism stability in 2 patients, and a MAF increase in 1 patient (from 1.9% to 5%). TGPs identified 4/4 cases of mosaicism whilst WES detected only 1/3.

**Discussion:**

Somatic *NLRP3* mosaicism was present in 40% of this paediatric cohort, confirming the key role of this phenomenon in disease pathogenesis and in genetic confirmation of CAPS diagnosis. MAFs can be extremely low, which warrants caution regarding lower detection limits of the sequencing techniques utilized. Mosaicism level may vary over time in some patients, with diagnostic and potential therapeutic implications.

## Introduction

Autosomal dominant (AD) gain-of-function mutations in the *NLRP3* gene are known to cause cryopyrin-associated periodic syndrome (CAPS). More recently, AD mutations in *NLRP3* have also been linked to non-syndromic sensorineural hearing loss DFN34 as well as to Keratoendotheleiitis fugax hereditaria (1).

The first description of somatic *NLRP3* mosaicism in a patient with chronic infantile neurological cutaneous articular syndrome (CINCA), also known as neonatal-onset multisystem inflammatory disease (NOMID) occurred almost 20 years ago (2). Since then, several studies (3–5) have confirmed the important role of mosaicism in *NLRP3* mutation-negative CAPS patients. These were prompted by the clinical observation that up to 50% of CAPS patients with identical clinical features and response to anti-IL-1 treatment, show no mutation in *NLRP3* by conventional DNA sequencing, suggesting the presence of either genetic heterogeneity, or low levels of somatic *NLRP3* mutations (2, 6, 7). Reported frequencies of *NLRP3* mosaicism in the literature vary from 10%–69% (3) of “mutation-negative” CAPS cases, whilst a more recent review established a general estimate of around 19% for *NLRP3* mosaicism (4).

The frequency of mutant cells can be quite low, even lower than 5% in blood, yet still cause disease. Therefore, the most significant limitation for an adequate genetic diagnosis is the requirement of methods sensitive enough to pick up mutations present at very low read frequencies with statistical confidence (8). Advances in next-generation sequencing (NGS) methods such as targeted gene panels (TGP) with high depth coverage or amplicon-based deep sequencing (ADS) made these the methods of choice to detect somatic mosaicism by several groups (7–9). Advances in ADS technology could increase the success rate of genetic diagnosis for mutation-negative CAPS from 60 to 80%, which would greatly benefit the care of these patients by increasing diagnostic yield and help prevent potentially irreversible complications by allowing earlier targeted treatment with IL-1 blockade (8).

Although the phenotype of patients with somatic *NLRP3* mosaicism is very typical of the disease, a few studies tried to ascertain if there were any differences in relation to CAPS patients with germline mutations (3, 9). Thus, patients with CINCA/NOMID and *NLRP3* mosaicism were found to have a lower incidence of intellectual disability, and milder neurological symptoms overall following genotype matched comparison. One possibility to explain this could be the level of mosaicism in central nervous system cells or glial cells (3). Regarding Muckle-Wells Syndrome (MWS), the differences between patients with germline and mosaic mutations were slightly broader. MWS patients with mosaicism seemed to have a reduced incidence of AA-type amyloidosis when compared to patients with germline mutations, an increased incidence of recurrent arthritis, as well as older ages at the onset of the disease and that of sensorineural deafness. Moreover, absence of family history is a requisite in patients with mosaicism, whilst patients with germline mutations may have affected first-degree relatives. However, one of the most important and impactful differences was the significant delay in obtaining access to treatment with anti-IL1 drugs in patients with mosaicism, which was only achieved once the molecular diagnosis of mosaicism was secured (9).

The differences in clinical severity between mosaic and germline CAPS patients can be due to several factors, including the type of amino acid exchange, its location in the cryopyrin protein, the functional consequence of the mutation, as well as the level (percentage of cells) and tissue distribution of the mosaicism (7, 9). Previous mosaicism studies in paediatric patients with *NLRP3* mosaicism showed presence of the mosaic mutation in comparable levels both in the myeloid and lymphoid lineages as well as affecting cells from ectodermic origin (3, 7, 10), unlike what has been described in adult late-onset CAPS patients (11–13).

In this study, we assessed the possibility of somatic mosaicism in a cohort of paediatric patients from a single UK centre with a clinical diagnosis of CAPS and no *NLRP3* mutations identified through conventional DNA sequencing. In identified cases, additional mosaicism studies focused on tissue distribution, stability over time, genotype-phenotype correlation, and possibility of transmission to offspring. Given the difficulty of detecting low-level mutations, we have also tried to compare different sequencing methods regarding the ability for detecting somatic mosaicism.

## Methods

### Ethics compliance

This study had the full approval of the medical ethics committee of Great Ormond Street Hospital NHS Foundation Trust (GOSH) (REC reference 08/H0713/82). Fully informed written consent was obtained from the patient's parents in accordance with the Declaration of Helsinki, and written assent where age appropriate.

### Patients

Patients were recruited from GOSH. Inclusion criteria were a typical phenotype of CAPS according to the Eurofever/PRINTO clinical classification criteria for CAPS (14), good response to treatment with IL-1 blockade (normalization of CAPS-DAS scores and inflammatory markers), and a previous Sanger sequencing of the *NLRP3* gene (either in its entirety or at least of exons 3, 4 and 6 in transcript NM1243133.1 (now known as exons 4, 5 and 7 respectively in the new updated transcript NM_001243133.2) (15) compatible with wild-type allele. Data on patient demographics, clinical presentation, and response to treatment were collected from clinical records.

### DNA extraction

Genomic DNA was extracted using the kits listed below according to the manufacturers' instructions: DNA from whole blood was extracted using the QIAamp DNA Blood Mini kit (Qiagen, Germany); DNA from hair/nails and leukocyte sub-populations was extracted using the QIAmp DNA Investigator Kit (Qiagen, Germany); DNA from saliva was extracted using the Oragene DNA Saliva Self-collection Kit (DNA Genotek, Canada); DNA from buccal cells was extracted using the Gentra Puregene Buccal Cell Kit (Qiagen, Germany); DNA from urine samples was extracted using the Norgen Urine DNA Isolation Kit for exfoliated cells (Norgen Biotek, Canada).

### Leukocyte subpopulations isolation from peripheral blood

Purification of different leukocyte subpopulations was performed by staining with monoclonal antibodies (mAb) anti-CD19 (B cells), CD3 (T cells), CD16, and CD56 (NK cells), CD14 (monocytes; Becton Dickinson or Biolegend), and cell sorter technology (FACS ARIA; BD Biosciences, USA).

### Genetic studies

The TGPs used in this study were the Vasculitis and Inflammation panel (16) (VIP), neuroinflammation panel (17) (NIP) and the NHS England autoinflammation panel (18) (from here on designed as AIP) as previously described. Whole-exome sequencing (WES) library preparation was performed with the Ilumina Nextera Rapid Capture Exome Library Preparation Kit according to manufacturer's instructions and sequencing in Hiseq 1,000 or NextSeq 500 platforms. Reads were aligned to GRCh38 using Burrows-Wheeler Aligner (19), annotation was performed using wANNOVAR (20) and variants were filtered in-house. ADS was performed in an IonTorrent PGM platform using the Ion Torrent PGM HiQ Sequencing kit as previously described (12).

### Funding

This work has been partially funded by PID2021-125106OB-C31 (JIA) grant from the Ministerio de Ciencia e Innovación (MCIN) | Agencia Estatal de Investigación (AEI) | 10.13039/501100011033 | Fondo Europeo de Desarrollo Regional (FEDER), UE. This work was also funded in part from institutional grants from Cromwell Hospital and supported by the National Institute for Health Research Biomedical Research Centre at Great Ormond Street Hospital for Children NHS Foundation Trust and University College London.

## Results

Ten patients with a clinical diagnosis of CAPS who were *NLRP3* mutation-negative by conventional Sanger sequencing were recruited to this study. Six patients had a CINCA/NOMID phenotype, whilst four had a MWS phenotype. Onset of symptoms occurred during the first year of life in all patients. Male sex was over-represented in this cohort with only two female patients. Patient 5 was found to have the germline p.G779V *NLRP3* mutation, which was previously missed due to a restrictive Sanger approach that did not include exon 5 (exon 6 in the new updated transcript NM_001243133.2) (14). Patient 6 had the germline p.D512Y mutation in the *NOD2* gene and in retrospect the phenotype was compatible with Blau syndrome.

### Amplicon-based deep sequencing results

Four out of 10 patients were identified as having somatic *NLRP3* mosaicism (Table 1). The whole cohort can be seen in Supplementary Table S1. The onset of symptoms ocurred during the first months of life in all patients with somatic mosaicism and mean age at clinical diagnosis was 3.4 years (range: 1–5.6 years). All four cases had a very typical CAPS phenotype and response to treatment with IL-1 blockade (with normalization of CAPS-DAS scores and inflammatory parameters). Patients 1 and 3 had a CINCA/NOMID phenotype, and Patients 2 and 4 had a MWS phenotype. All mosaicism cases were male. Clinical characteristics of mosaic patients are depicted in Table 2. As usual in children with CINCA/NOMID, Patient 1 required an increased dose of canakinumab (8 mg/kg/4-weekly, subcutaneously) to control his symptoms.

**Table 1:** Mosaicism studies.

Patient	Nt change	Aa change	% mutated allele									
			Blood	Neut	Mono cytes	B cells	T cells	NK cells	Saliva	Buccal swab	Urine	Hair/nails
1—♂, 7y	c.1698 C > A	p.F566L	14.5	ND	ND	ND	ND	ND	ND	ND	ND	ND
2—♂, 5y	c.1699 G > A	p.E567K	3.1	ND	3.4	3.9	2.9	3.8	3.9	2.0	1.7	0.0/0.0
3—♂, 1y	c.1691 G > A	p.G564D	12.5	12.2	11.9	12.5	14.1	11.8	ND	ND	ND	ND
4—♂, 7y	c.920 G > T	p.G307V	2.5	ND	ND	ND	ND	ND	1.5	0.0	0.0	0.0/0.0

Nt, nucleotide; Aa, amino acid; y, years; neut, neutrophils; NK, natural killer; ND, not done, ♂, male; ♀, female; y, years.

**Table 2:** Clinical presentation of patients with NLRP3 mosaicism.

Patient	CAPS subtype	Age at onset	Fever	Urtic rash	Headaches	Fatigue	Cold trigger	Arthritis	Eye involvement	SN deafness	Aseptic meningitis	Frontal bossing	Mid facial hypoplasia	Acute phase reactants	AA Amyloidosis/ nephrotic syndrome
1-♂ 7y	CINCA/NOMID	birth	+	+	+	+	−	+ BO	−	−	+	+	+	+	−/−
2-♂ 5y	MWS	1st week	−	+	+	+	−	+	Conjunctivitis coloboma	−	+	+	−	+	−/−
3-♂ 1y	CINCA/NOMID	birth	+	+	+	+	−	−	papilledema	−	+	+	−	+	−/−
4-♂ 7y	MWS	6 months	−	+	+	+	+	+	conjunctivitis	−	−	−	−	+	−/−

MWS, muckle wells syndrome; BO, bony overgrowth; SN, sensorineural; CNS, central nervous system; ICP, intracraneal pressure; HC, head circumference; ♂, male; ♀, female; y, years; CINCA, chronic infantile neurological cutaneous and articular syndrome.

Four different mutations were identified in these patients with MAFs ranging from 3.1% to 14.5% (Table 1). In Patient 1 the c.1698C > A transversion was identified, resulting in the missense p.F566L mutation, with a MAF of 14.5% (mean coverage 1151x). This mutation was initially identified by WES as previously reported (21). In Patient 2 the c.1699G > A transition resulting in the missense p.E567K mutation was detected with a MAF of 3.1% (mean coverage 755x). Regarding Patient 3, the c.1691G > A transition was identified, resulting in the missense p.G564D mutation, with a MAF of 12.5% (mean coverage 710x). Finally, in Patient 4, the c.920G > T transversion was identified, resulting in the missense p.G307V mutation with a MAF of 2.5% (mean coverage 7763x). Interestingly, this latter mutation had been initially missed due to a extremely low MAF. As shown in Supplementary Table S1, two of these mutations, p.F566L and p.E567K, had already been previously described (3), albeit only in the somatic form. The third, p.G564D, was novel at the time but has since been described also in somatic form by Rowczenio et al. (13). The fourth mutation, p.G307V, had already been described in germline and mosaic forms in patients with severe forms of CINCA/NOMID (3, 22).

### Cellular mosaicism studies

Evaluation of mosaicism distribution in isolated leukocyte subpopulations (Table 1) revealed similar allelic frequencies in myeloid and lymphoid cells for Patients 2 and 3, which suggests that the mutation arose before the stage of differentiation into common myeloid and lymphoid progenitors. The lower allelic frequencies detected in other tissues such as saliva, buccal swab and urine are more likely to reflect leukocyte contamination of these samples, rather than the mutation affecting other embryonic layers, since it was absent in DNA extracted from hair and/ or nails (ectodermal origin), however the latter cannot be excluded.

### Mosaicism frequency over time

DNA extracted from blood samples obtained at different time points with at least one year interval was available for three patients. Interestingly, in patient 4, a very small increase in the allelic frequency of the somatic mutation over time (see below) had important consequences for mosaicism detection and diagnosis. In the other two it remained stable (mean time interval-1.8 years; range: 1–6 years) (Table 3).

**Table 3:** Variation in mosaicism frequency over time. Results by ADS for Patients 2 and 3 and by different sequencing methods for Patient 4. Read coverage for the targeted panels in patient 4 is presented as reads with the mutation/ total read coverage at that genomic location (similar data for ADS experiments is presented in Supplementary Table S2).

Patient (gender; age)	Mutation	MAF Time point 1 (read coverage)	MAF Time point 2 (read coverage)	Interval (years)
2-♂, 7y	p.E567K	3.1%	3.5%	1
3-♂, 1y	p.G564D	11.8%	12.5%	1
4-♂, 7y	p.G307V	1.9% ADS* (mean 4,604) 3% NIP (7/235) ND—VIP (0/35) 2% AIP (38/1,910)	2.5% ADS (mean 7,763) 5% NIP (9/177) 4% VIP (8/210) 3% AIP (42/1,402)	6

MAF, mutant allele frequency; ADS, amplicon-based deep sequencing; NIP, neuro-inflammation panel; VIP, vasculitis and inflammation panel; AIP, autoinflammation panel; ND, not detected.

* Previously undetected, ♂- male; ♀-female; y- age in years at study enrollment.

Patient 4 had a very small increase in allelic frequency from 3 to 5% over a 6-year period (results by targeted gene panel—NIP) finally enabling a genetic diagnosis, as this mutation had been initially missed by ADS, targeted panel (VIP) and WES. However, the discovery of the p.G307V mutation at an allelic frequency of 4% on VIP prompted reanalysis of DNA samples from both time points by three different targeted panels as well as ADS. All methods confirmed the presence of this somatic mutation in DNA samples from time point 2, but it was necessary to re-analyse the sample by ADS and/or review the read alignment manually for the DNA sample from time point 1 (AIP panel), as the mutation's allelic frequency was extremely low and otherwise discarded as a probable false positive (allelic frequency range: 1.9%–3%). The fact that the somatic mutation was not detected on the time point 1 sample by VIP is likely related to the low read coverage on that specific genomic location (Table 3).

### Comparison of sequencing methods for detection of somatic mosaicism

To try to ascertain which sequencing methods were effective in detecting somatic mosaicism, a comparison of the results obtained by different methods was performed as shown in Table 4.

**Table 4:** Comparison of sequencing results in paediatric somatic NLRP3 mosaicism CAPS cases.

Patient (gender, age)	Phenotype	Aa change	% mutated allele	Sanger detection	ADS detection	TGP detection	WES detection
1-♂, 7y	CINCA	p.F566L	14.5	No	Yes	Yes	Yes
2-♂, 5y	MWS	p.E567K	3.1	No	Yes	Yes	No
3-♂, 1y	CINCA	p.G564D	11.8	No	Yes	Yes	N/A
4-♂, 7y	MWS	p.G307V	1.9*–5	No	Yes (2nd time point)	Yes (2nd time point)	No

CAPS, cryopyrin associated periodic syndromes; MWS-,muckle-wells syndrome; CINCA, chronic infantile neurologic cutaneous articular syndrome; ADS, amplicon-based deep sequencing; TGP, targeted gene panel; WES, whole exome sequencing; N/A, not available.

* Extremes of variation between two time-points.

There was no difference between ADS and a TGP regarding mosaicism detection in this cohort, as these were the only methods able to detect all cases. More specifically in Patient 4, detection of the somatic *NLRP3* mutation through both methods was only possible on the second time-point, when the allelic frequency of the mutation was higher than 2%–3%. Mosaicism detection by WES was only possible in Patient 1 who had the highest percentage of mutated allele of this cohort. WES data were not available for Patient 3, however an allelic frequency of 11.8% is considered above the threshold of detection for this method (21).

## Discussion

Of this paediatric UK cohort of 10 patients who were initially considered as “mutation negative CAPS”, one patient was found to have a *NOD2* mutation and an eventual diagnosis of Blau syndrome; one patient had a germline mutation in *NLRP3* that was missed by an historic Sanger sequencing approach that only focused on exons considered to harbour mutation hotspots; and *NLRP3* mosaicism was present in 4/10 (40%), confirming the important role of somatic *NLRP3* mosaicism some CAPS patients. Thus, a confirmatory genotype was identified in 60% in this “mutation negative” series.

Two studies have been pivotal to the understanding of somatic mosaicism in CAPS by establishing evidence of unification of the intracellular and extracellular inflammatory cascades that results in amplification of the inflammatory response, thus explaining why even only a small proportion of mutated leucocytes can result in severe systemic inflammatory disease (23–25). It has been demonstrated that extracellular specks released during pyroptosis can act as an initial danger signal and bypass the sensor-activating portion of the inflammasome cascade, as well as recruit NLRP3 and caspase-1, thus acting as a functional extracellular inflammasome (23–25). Extracellular ASC specks have also been identified in the serum of patients with somatic *NLRP3* mutations (23). Together, these reports demonstrate how mutations in only 4% of leukocytes could lead to a full-blown severe CAPS phenotype (25), since it is now apparent that the biology of the inflammasome is not restricted to the intracellular compartment.

The p.F566L mutation identified in Patient 1 has been previously reported in 2 unrelated patients with CINCA/NOMID syndrome in mosaic state with estimated allelic mosaicism of 11.5% and 14.6%, respectively (3). The p.E567K *NLRP3* mutation found in Patient 2 has been previously described in a patient with MWS with an estimated mosaicism of 6.5% (26). Functional studies performed with these two variants confirmed their suspected pathogenicity through induction of THP-1 necrosis-like programmed cell death and ASC-dependent NF-kB activation (3, 26). The p.G564D mutation identified in Patient 3 has also been described in somatic form in a patient with late-onset CAPS and its pathogenic role was confirmed by functional studies showing increased ASC aggregates in patient's serum (13). The p.G307V mutation in Patient 4 had also been previously described in a CINCA/NOMID patients as either germline (22) or mosaic (3). Therefore, all these mutations fulfil several categories of evidence of pathogenicity according to the guidelines of the American College of Genetics and Genomics/ American College of Pathology for gene variant classification (27), as seen in Supplementary Table S1.

In this cohort we found mutations thus far only described in a somatic form and others described as germline in a more severe (CINCA/NOMID) form. This seems to support the notion that some mutations are so damaging that may be incompatible with life in a germline form, and that somatic mutations tend to be milder than if occurring as germline. Therefore, the clinical severity of a somatic *NLRP3* mutation would be a function not only of its allelic frequency, but also of its impact on the function of the cryopyrin protein (9).

To assess the distribution of mosaicism, we selected samples representative of the three embryonic layers: blood and saliva (mesoderm), buccal swab, hair root and nails (ectoderm) and urine (endoderm/mesoderm) (28). As expected, a slightly different pattern was noted between this paediatric cohort and the adult late-onset patients reported in the literature. In late-onset adult patients, increased allelic frequencies were found in the myeloid compartment in comparison to the lymphoid compartment, inclusively with significant enrichment in the former seen in some patients, with relatively small allelic frequencies in other samples likely corresponding to leukocyte contamination. This distribution places the mutational event at the level of either a common myeloid progenitor or a pluripotent hematopoietic stem cell. In late-onset CAPS, a possible explanation to at least partially account for the difference between myeloid and lymphoid cell lineages could be an impairment of differentiation of the lymphoid lineage, with selective increase of the myeloid lineage, which has been known to occur in the elderly (12). In this paediatric, early-onset cohort, however, this was not the case: in our patients, similar allelic frequencies were found between myeloid and lymphoid cells.

It is important to note that in cases of mosaicism presenting in the first weeks of life, in theory, the mutational event could have occurred at any stage, from early embryonic development until a mutation occurring in a myeloid progenitor or pluripotent hematopoietic stem cell at birth or in the first weeks of life. In Patient 4 the somatic mutation was not detected in other tissues (buccal swab, hair, nails and urine) therefore likely placing the occurrence of the mutational event at the level of a pluripotent haematopoietic stem cell. In Patient 2, the presence of lower allelic frequencies of the somatic mutation in buccal cells and urine could likely derive from leukocyte contamination of the samples, although an earlier occurrence of the mutational event cannot be ruled out at this point. This could have practical clinical consequences for this patient, because if the mutation has also affected the gonadal tissue, it could potentially be transmitted to offspring in germline form.

We have confirmed that even very low levels of *NLRP3* mosaicism may cause CAPS (the lowest being 1.9% in one case) as described previously (10). Patient 4 had a slight rise in mosaicism which eventually enabled the diagnosis, but without any obvious clinical impact to phenotype or therapeutic response. It is therefore possible that some patients who are *NLRP3* mutation-negative even after investigation for somatic mosaicism, may actually have a mutation with extremely low frequency which could be under the threshold for detection of past (or current) methods. Thus, repeating a deep sequencing method at a later point in such patients may be warranted, either with a more sensitive method or to look for an increase in allelic frequency above the threshold for detection. However, significant increases in mosaicism frequency over time might have impact on treatment response as reported by Rowczenio et al. (13), although it remains to be seen if this will occur in this paediatric cohort. It has also been suggested that IL-1 blockade might also influence mosaicism levels. However, what has been documented thus far is a decrease in mosaicism levels following anti-IL-1 treatment (10, 12), probably due to treatment reducing the increased haematopoiesis caused by the IL-1 overproduction and consequently normalizing the total white cell and neutrophil counts.

The difficulties in detecting the somatic *NLRP3* mutation in paediatric Patient 4 highlight the importance of choice of sequencing method as well as existing limitations despite the fantastic advances in NGS technologies in recent years. The comparison of results of different sequencing methods on mosaicism detection showed that TGPs appear to offer the optimal compromise between breadth and depth for the genetic diagnosis of these patients, as they can simultaneously identify mutations in several genes even at very low-levels of mosaicism. Genetic panels are now currently used as standard practice in many genetic laboratories, although many still use restrictive Sanger approaches. However, even in those using NGS gene panels, a survey found that only 17% of respondent laboratories were including routine testing for somatic mosaicism in their practice (29), even though consideration of low-level somatic mosaicism has been included in recent guidelines for best practice in the genetic diagnosis of autoinflammatory diseases (30). Furthermore, to the best of our knowledge, there is only one published study comparing sequencing methods in autoinflammation, which compared the effectiveness of a TGP to Sanger sequencing in the diagnosis of autoinflammatory diseases (31). Nevertheless, the tendency in recent years has been towards favouring methods with higher breadth.

In this series, if WES had been the only method used, at least half of this cohort would have remained without a genetic diagnosis due to comparative lack of sensitivity for the detection of low-level mosaicism. An interesting question in this regard would be to see if whole-genome sequencing (WGS) could offer advantages over WES. Two of our patients were previously recruited to WGS studies, however, in both cases there was no evidence of the presence of mosaicism in WGS data, even after careful manual review of the read alignment at the given genomic location (data not shown). Somatic mutations with an allelic frequency of 20% can be described as low-level in WGS projects (32), therefore it is not entirely convincing that even the mosaicism case with an allelic frequency of the mutation of 14.5% would be easily detected by WGS**.**

There are several still unanswered but potentially relevant questions to be addressed: namely the distribution of somatic mosaicism in additional non-haematopoietic tissues and different leukocyte populations; additional time points to assess mosaicism stability over time; and to assess the possibility of vertical transmission of the mutated allele to offspring.

## Conclusion

The work described herein confirmed the importance of somatic *NLRP3* mosaicism in the genetic diagnosis of paediatric cases of “mutation-negative” CAPS from a single centre UK cohort. Furthermore, mosaicism levels may vary over time in some patients and could be picked up later in life if repeat testing is undertaken. The comparison of sequencing methods suggests that TGPs may offer an advantage in this setting, whilst the diagnostic yield for mosaicism detection using WES or WGS may be lower.

## Data Availability

The original contributions presented in the study are included in the article/supplementary material, further inquiries can be directed to the corresponding author/s.
