# Supplementary material for: Somatic NLRP3 mosaicism in patients with “mutation-negative” CAPS: insights from a single centre UK cohort
Source: Front Pediatr. 2025 Jun 5;13:1598748. doi: 10.3389/fped.2025.1598748 (PMC12176735; doi:10.3389/fped.2025.1598748)
Supplement: Supplementary file 1 [file Table1.docx]

**Supplementary Table 1: Results of amplicon-based deep sequencing.** Values presented as mean; analyses performed in triplicate. Where no somatic mutation was identified, the read coverage reflects the mean coverage of NLRP3 exons. Where a somatic mutation was identified, the read coverage pertains to the genomic location of the somatic mutation; #- previously undetected; ^1^– results obtained by Whole Exome Sequencing; ^2^- NLRP3 mosaicism not detected; aa– amino acid; HET- Heterozygous; CINCA Chronic infantile neurological cutaneous articular syndrome; MWS- Muckle Wells Syndrome;♂ - male**; ♀-**female¸ y- years; ACMG/AMP- American College of Medical Genetics and Genomics/ American College of Pathology; PS2-De novo (both maternity and paternity confirmed in a patient with the disease and no family history: PS3- well-established in vitro or in vivo functional studies supportive of a damaging effect; PM1- located in a mutational hotspot and/or critical and well-established functional domain; PM2- absent from control databases; PP3- multiple lines of computational evidence are supportive of a deleterious effect

| **Patient** | **Phenotype** | **Nucleotide change** | **Aa change** | **% mutated allele** | **Mean Coverage** | **ACMG/AMP classification** | **Reference** |
| --- | --- | --- | --- | --- | --- | --- | --- |
| 1**- ♂**, 8y | CINCA | **c.1698 C>A** | **p.F566L** | **14.5** | 1151x | PS2, PS3, PM1, PM2, PP3 | Tanaka *et al.*, 2011 |
| 2- **♂**, 8y | MWS | **c.1699 G>A** | **p.E567K** | **3.1** | 1489x | PS2, PS3, PM1, PM2, PP3 | Tanaka *et al.*, 2011 |
| 3- **♂**, 1y | CINCA | **c.1691 G>A** | **p.G564D** | **11.8** | 710x | PS2, PS3, PM1, PM2, PP3 | Rowczenio *et al*., 2017 |
| 4- **♂**, 8y | MWS | -^#^/ **c.920G>T** | -^#^/ **p.G307V** | **-**^#^ /**1.9** | 3517x/4604x | PS2, PS3, PM1, PP3 | Matsubara *et al*., 2006 |
| 5- **♂**, 2y | CINCA | c.2336G>T | p.G779V^1^ | HET | 1655x | PS2, PM2, PP3 |  |
| 6**-♀**, 16y | CINCA-like | -^2^/*NOD2* c.G1534T | -^2^ / *NOD2* p.D512Y*^1^* | - /HET | 760x |  |  |
| 7- **♂**, 14y | MWS | - | - | - | 1008x |  | **2- ♂, 12y** |
| 8**-♀**, 2y | CINCA-like | - | - | - | 1655x |  |  |
| 9- **♂**, 6y | CINCA-like | - | - | - | 2316x |  |  |
| 10**- ♂**, 7y | MWS | - | - | - | 760x |  |  |
