# Supplementary material for: Somatic NLRP3 mosaicism in patients with “mutation-negative” CAPS: insights from a single centre UK cohort
Source: Front Pediatr. 2025 Jun 5;13:1598748. doi: 10.3389/fped.2025.1598748 (PMC12176735; doi:10.3389/fped.2025.1598748)
Supplement: Supplementary file 2 [file Table2.docx]

**Supplementary Table 2: Read coverage for ADS mosaicism experiments**. The read coverage is presented as reads with the mutation/total number of reads at that genomic location (values expressed as mean; analyses performed in triplicate). (ADS- amplicon-based deep sequencing; Nt- nucleotide; Aa- amino acid; Neut- neutrophils; NK- natural killer; ND- not done.).*-after revision, initially not detected.

| **Patient** | **Nt change** | **Aa change** | Read coverage | | | | | | | | | | |
| --- | --- | --- | --- | --- | --- | --- | --- | --- | --- | --- | --- | --- | --- |
|  |  |  | **Blood**  **Time**  **point 1** | **Blood Time point 2** | **Neut** | **Mono**  **cytes** | **B cells** | **T cells** | **NK cells** | **Saliva** | **Buccal swab** | **Urine** | **Hair / nails** |
| 1 | c.1698  C>A | p.F566L | 167/1151 | ND | ND | ND | ND | ND | ND | ND | ND | ND | ND |
| 2 | c.1699 G>A | p.E567K | 46/1489 | 126/3584 | ND | 47/1385 | 35/891 | 36/1250 | 42/1111 | 85/2189 | 31/1539 | 13/778 | 0/944 |
| 3 | c.1691  G>A | p.G564D | 84/710 | 487/3896 | 695/5698 | 303/2545 | 441/3527 | 662/4699 | 647/5486 | ND | ND | ND | ND |
| 4 | c.920 G>T | p.G307V | 87/4604* | 204/7763 | ND | ND | ND | ND | ND | 147/9331 | 0/3070 | 0/537 | 0/2775 0/217 |
|  |  |  |  |  |  |  |  |  |  |  |  |  |  |
